# Rational Primer and Probe Construction in PCR-Based Assays for the Efficient Diagnosis of Drifting Variants of SARS-CoV-2

**DOI:** 10.1155/2022/2965666

**Published:** 2022-05-13

**Authors:** Divya RSJB Rana, Nischal Pokhrel, Santosh Dulal

**Affiliations:** ^1^Hari Khetan Multiple Campus, Tribhuvan University, Birgunj, Nepal; ^2^B and B Medical Institute, Lalitpur, Nepal; ^3^Department of Natural and Applied Sciences, Nexus Institute of Research and Innovation (NIRI), Lalitpur, Nepal

## Abstract

The genome sequence of severe acute respiratory syndrome coronavirus 2 (SARS-CoV-2) has been evolving via genomic drifts resulting in “emerging/drifting variants” circulating worldwide. The construction of polymerase chain reaction (PCR) assays for the reliable, efficient, and specific diagnosis of the drifting variants of SARS-CoV-2 is specifically governed by the selection and construction of primers and probes. The efficiency of molecular diagnosis is impacted by the identity/homology of the genome sequence of SARS-CoV-2 with other coronaviruses, drifting variants or variants of concern (VOCs) circulating in communities, inherent capacity of mutation(s) of various target genes of SARS-CoV-2, and concentration of genes of interest in host cells. The precise amplicon selection and construction of primers and probes for PCR-based assays can efficiently discriminate specific SARS-CoV-2 drifting variants. The construction of single nucleotide polymorphism (SNP)-specific primers and probes for PCR assays is pivotal to specifically distinguish SARS-CoV-2 variants present in the communities and contributes to better diagnosis and prevention of the ongoing COVID-19 pandemic. In this study, we have utilized *in silico*-based bioinformatic tools where the alignment for genes, the positions and types of SNPs/mutations of VOCs, and the relative number of SNPs per nucleotide in different genomic regions were investigated. Optimal and specific genome region (amplicon) selection with comparatively lower mutability in the SARS-CoV-2 genome should be prioritized to design/construct PCR assays for reliable and consistent diagnosis in various regions of the world for a longer duration of time. Further, the rational selection of target genes that is at an optimal detectable concentration in biological samples can bolster PCR assays of high analytical sensitivity. Hence, the construction of primers and probes with the rational selection of targeting specific E gene, genomic regions with highly conserved sequences, multiple target genes with relatively lower mutability and detectable level of concentration, SNP-specific binding regions of spike (S gene) protein, and shorter amplicon size (100–150 bp) are vital for the PCR assays to achieve optimal efficiency in the point-of-care laboratory diagnosis of circulating drifting variants of SARS-CoV-2 with optimal accuracy.

## 1. Introduction

Cutting-edge molecular assays, specifically quantitative polymerase chain reaction (qPCR), have proved to be a crucial technology to give high sensitivity and specificity for the diagnosis since the early days of coronavirus disease 2019 (COVID-19) emergence [1]. Along with real-time reverse transcription polymerase chain reaction (rRT-PCR), other tests have been designed for the diagnosis of SARS-CoV-2 including serological lateral flow assay test for antigens and/or antibodies, enzyme-linked immunosorbent assay (ELISA), and reverse transcription loop-mediated isothermal amplification (RT-LAMP) [2–4]. In the initial days, clinical diagnosis was even relied upon, in addition to, less specific computed tomography (CT) scans [5]. The laboratory diagnosis of SARS-CoV-2 is a time-bound process as it is highly dependent upon the viral load of the patient, which can fluctuate along the duration of the disease [2]. Among these entire tests, RT-PCR can provide high sensitivity and specificity for the longest duration of disease state, i.e., from the disease incubation phase till recovery phase [2]. The rRT-PCR is considered as a gold standard for SARS-CoV-2 diagnosis and has continuously been used widely in public health/diagnostic laboratories from resource-constrained/limited to well-resourced settings in the fight against the ongoing COVID-19 pandemic. Whole-genome sequencing (WGS), an invaluable tool for genomic surveillance, has helped to identify novel infections [6] and variants and develop diagnostic kits for rapid detection and outbreak containment [7]. However, it is still not applicable for the point of care for routine laboratory diagnosis in poor/limited resourced settings due to costly next gene sequencing (NGS) machine, reagents, and consumables, requiring well-equipped and highly sophisticated laboratory and well-trained/skilled human resources. Approximately 4.3 billion tests have been performed with 287 million confirmed positive cases worldwide for 7.8 billion world population by the end of December 2021 [8].

Coronaviruses, or Nidovirales order in general, have the largest RNA genomes of all RNA viruses known, and due to high-fidelity RNA replication and transcription machineries, the number of new mutations occurring per replication is relatively low (S G) [9] in comparison with other RNA virus such as orthomyxovirus. However, a large population of infected individuals and the selective pressure of evading the immune system and having a better target cell attachment factors have given rise to new variants of concerns (VOCs), such as Alpha, Beta, Gamma, Delta, and Omicron variants. These variants have accumulated a considerable number of mutations, especially in their structural genes such as S and N, and as a result, some variants such as Omicron variant may not be detectable by currently available diagnostic tests due to S gene target failure or S gene dropout [10]. Due to resource constraints, commercial diagnostic kits that detect only one to two genes have also come to be used [11], and this may increase the chance of false-negative results in case qPCR fails to diagnose one or two genes.

Studies have reported variable sensitivities for the rRT-PCR assays used to diagnose SARS-CoV-2 [12,13]. Although test sensitivity could be lowered by errors in methodology, instrument, and diagnostic kits, a decrease in PCR efficacy due to mutation in the primer or probe binding sites is very hard to account for [12] unless sequencing of the PCR-targeted genomic region is carried. Primer and probe design plays particularly important roles to ensure target detection and quantification, replicate accuracy, sensitivity, and amplification efficiency of all targets, and reduce variant-specific cross-reactivity. Each primer and probe set needs to be evaluated on an individual basis (reference genes and genes of interest) to determine designs and conditions that are ideal for the target amplification via standard curve methodology with dilution series to achieve close to 100% efficiency. The combination of proper functional validation primers and probes in a reaction is also desired as different primer pairs and/or probes of combination in a reaction should not interact with each other.

The prime objectives of the PCR assays are to provide a reliable diagnosis for a longer duration of time during the evolution/mutations of the virus with optimal accuracy. Currently available commercial molecular rRT-PCR kits have lower efficiencies and/or inefficient to detect/differentiate the drifting variants of SARS-CoV-2 [10]. Primers and probe design/construction are arguably the most crucial factor in a multiplex assay where more than one target is analyzed in the same real-time PCR. Therefore, the construction of primers and probes based on the latest genetic information/data of emerging SARS-CoV-2 variants/drifting variants is desired to enhance real-time PCR efficiency and the optimal accuracy necessary to discriminate variants for COVID-19 diagnosis.

This bioinformatic study provides the rational primer and probe construction techniques/deep understanding of PCR-based assays for the reliable detection of SARS-CoV-2 variants ensuring optimal accuracy and replicability.

## 2. Methods

*In silico*-based bioinformatic assessments were performed comparing SARS-CoV-2 Wuhan-Hu-1 (reference genome) to VOCs (Alpha, Beta, Gamma, Delta, and Omicron), bat SARS-like coronavirus, SARS-CoV, and bat coronavirus. Whole-genome sequence (WGS) or specific gene sequence data of SARS-CoV-2 were collected from NCBI Nucleotide GenBank and http://www.GISAID.org. MUltiple Sequence Comparison by Log-Expectation (MUSCLE) [14] was used for multiple sequence alignment (MSA) among genes of interest or gene fragments by MEGA11 software version 0.1. MUSCLE claims to achieve relatively higher average accuracy and better speed than ClustalW2 or T-Coffee, on the chosen options [14]. Positions and types of SNPs or mutations for variants of SARS-CoV-2 are described as specified in the GISAID and NCBI GenBank databases.

We have used supplementary data provided by Mercatelli et al. [15] for the analysis of mutations in various target genes to aid in the selection of specific primers and probes for better diagnosis of SARS-CoV-2 variants. Sample (SARS-CoV-2 genomes) extracted from http://www.GISAID.org exhibited their distribution and size (*N* = 48635) according to regions that are presented in Table 1. The sampling distribution has been skewed towards more developed regions of the world. Two-third of samples were contributed from Europe, one-third of samples jointly were contributed from North America, Asia, and Oceania, and the rest (2% sample) were contributed from Africa and South America. We also included 0.02% sample (11 genomes) with no origin of sample submission (GISAID.org) for our analysis, which does not necessarily impact our results/findings with this comparably minimal quantity of sample size (Table 1).

A preprint of the first draft of this study has previously been uploaded in bioRxiv [16] (https://www.biorxiv.org/content/10.1101/2021.04.04.438420v1.full.pdf), and an updated version is presented in this article. For the specific analysis of the collected genetic data, WPS Spreadsheet version 11 and GraphPad Prism 5 were utilized. We have calculated the relative number of SNPs per nucleotide in different genomic regions. Even though these data only include variant data as of June 2020, this analysis will help to understand the general trend of genes to mutate and give an idea about their relative mutability. We have determined unique mutation events in the genome of SARS-CoV-2, by grouping entries into “refpos,” “refvar,” and “qvar” categories in WPS Spreadsheet and by removing the entries with duplicate SNP variants. However, we have retained the SNP entries where the same nucleotide may have undergone different kinds of mutations.

To accurately quantify mutations in the 3′ UTR region, entries were removed for sequences corresponding to 3′ UTR at or before nucleotide 29674, i.e., the last nucleotide for ORF10, and only sequence from 3′ to ORF10 was included as true 3′ UTR. We also removed intergenic SNPs, which were 3 in total. We combined entries for NSP12a and NSP12b into NSP12, which corresponds to the RdRp gene. We calculated the number of mutations or SNPs, prevalent or unique, per nucleotide (Nt) per 10,000 genomes for each of the SARS-CoV-2 genes by dividing the number of SNPs in a given gene by product of nucleotide size of that given gene and number of genomes analyzed and then multiplying it by ten thousand. Prevalent mutation here includes all the mutations contained in each and every viral genome. The numbers were rounded off to two decimal places. Figures were drawn in GraphPad Prism v5 and WPS Excel. Gene coordinates of SARS-CoV-2 Wuhan-Hu-1 genome (NCBI GenBank Accession ID : NC_045512.2) were used as reference. As mutations of all kinds, sense or nonsense, impact PCR, mutations discussed here do not represent the evolutionary implications.

## 3. Results and Discussion

### 3.1. Selection of Target Genes for PCR Assays

Among the genes of choice used by WHO-collaborating laboratories, the Corman group (Charite, Germany) [1] used E gene and RdRp genes for the diagnosis of COVID-19, while other laboratories used N and/or ORF1 genes [17]. Commercially, the S gene has also been used [11]. “ORF1ab” has been frequently stated to be used for COVID-19 detection in the commercial PCR kits, but it is not clear whether it is the RdRp gene (NSP12). The first set of primers designed by the Corman group [1] selected RdRp and E gene as genes of choice. Though primers for N gene were also designed, the gene was abandoned due to low sensitivity during optimization. During the initial days of the pandemic, the exact strain of the virus was not confirmed and the Corman group recommended a set of primer for E gene, which could amplify the newly sequenced virus and similar SARS-like viruses and was considered a screening gene. This could be due to the relatively low synonymous and non-synonymous mutational differences in the SARS-CoV-2 virus compared with orthologous sequences from other bat and pangolin coronaviruses and SARS-CoV-2 [18], but this does not mean that the E (envelope) gene is the same in all of the related viruses.

We utilized MUSCLE, a high-throughput application for multiple sequence alignment, to achieve the highest score in sequence alignment and reductions in computational complexity [14].

**Figure 1:**
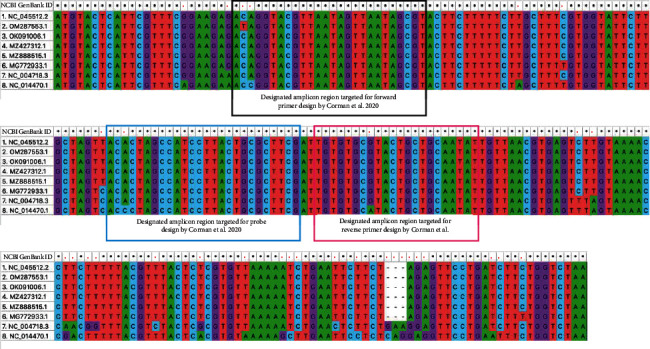
E gene multiple sequence alignment (MUSCLE) by MEGA11 version 0.1. The region of primers and probe was designed by [1]. Star (^*∗*^) signs denote perfect nucleotide position match, and red dots (.) denote mismatch. The black rectangle shape denotes the amplicon region for forward primer design, the blue rectangle shape denotes the amplicon region for probe design, and the pink rectangle shape denotes amplicon position for reverse primer position. Analyzed NCBI GenBank accession IDs for MSA for the entire E gene represent as follows: 1. NC_045512.2: E gene (nucleotide sequence: 26245–26472, 228 bp), SARS-CoV-2 isolate Wuhan-Hu-1, SARS-CoV-2 Ref. Genome; 2. OM287553.1: E gene (nucleotide sequence: 26170–26397, 228 bp), SARS-CoV-2/human/NLD/EMC-Omicron-1/2021; 3. OK091006.1: E gene (nucleotide sequence: 26218–26445, 228 bp) SARS-CoV-2/human/JPN/SARS-CoV-2, B.1.617.2 lineage, Delta variant/2021; 4. MZ427312.1: E gene (nucleotide sequence: 26214–26441, 228 bp), SARS-CoV-2/human/DEU/SARS-CoV-2_P.1 (Gamma)_VeroE6_210419_P3/2021; 5. MZ888515.1: E gene (nucleotide sequence: 26197–26424, 228 bp) SARS-CoV-2/human/THA/AFRIMS-COV0087/Alpha variant/2021; 6. MG772933.1: E gene (nucleotide sequence: 26150–26377, 228 bp), bat SARS-like coronavirus isolate bat-SL-CoVZC45; 7. NC_004718.3: E gene (nucleotide sequence: 26117–26347, 231 bp), SARS coronavirus Tor2; and 8. NC_014470.1: E gene (nucleotide sequence: 26018–26248, 231 bp), bat coronavirus BM48-31/BGR/2008.

**Figure 2:**
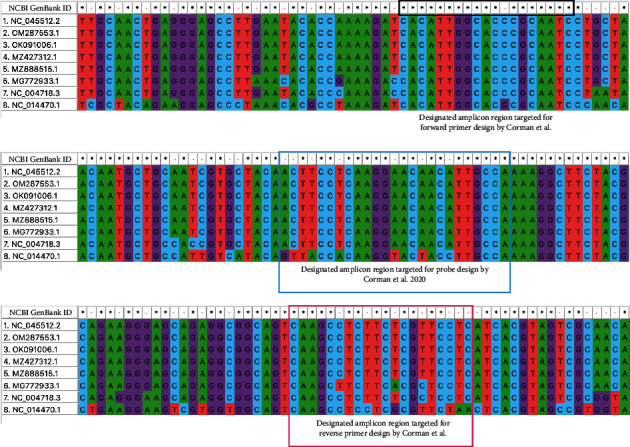
N gene designated fragment—MSA (MUSCLE) by MEGA11 version 0.1. The region of primers and probe is designed by [1]. Star (^*∗*^) signs denote perfect nucleotide position match, and red dots (.) denote mismatch. The black rectangle shape denotes the amplicon region for forward primer design, the blue rectangle shape denotes the amplicon region for probe design, and the pink rectangle shape denotes amplicon position for reverse primer position. Analyzed NCBI GenBank accession IDs for MSA for the entire E gene represent as follows: analyzed NCBI GenBank accession IDs for multiple sequence alignment for the N gene fragment represent as follows: 1. NC_045512.2: N gene designated fragment (nucleotide sequence: 28671–28850, 180 bp), SARS-CoV-2 isolate Wuhan-Hu-1, SARS-CoV-2 ref. Genome; 2. OM287553.1: N gene designated fragment (nucleotide sequence: 28587–28766, 180 bp), SARS-CoV-2/human/NLD/EMC-Omicron-1/2021; 3. OK091006.1: N gene designated fragment (nucleotide sequence: 28644–28823, 180 bp), SARS-CoV-2/human/JPN/SARS-CoV-2, B.1.617.2 lineage, Delta variant/2021; 4. MZ427312.1: N gene designated fragment (nucleotide sequence: 28644–28823, 180 bp), SARS-CoV-2/human/DEU/SARS-CoV-2_P.1 (Gamma)_VeroE6_210419_P3/2021; 5. MZ888515.1: N gene designated fragment (nucleotide sequence: 28622–28801, 180 bp), SARS-CoV-2/human/THA/AFRIMS-COV0087/Alpha variant/2021; 6. MG772933.1: N gene designated fragment (nucleotide sequence: 28576–28755, 180 bp), bat SARS-like coronavirus isolate bat-SL-CoVZC45; 7. NC_004718.3: N gene designated fragment (nucleotide sequence: 28520–28695, 180 bp), SARS coronavirus Tor2; and 8. NC_014470.1: N gene designated fragment (nucleotide sequence: 28059–28238, 180 bp), bat coronavirus BM48-31/BGR/2008.

We conducted MSA for the entire E gene (228–231 bp) (Figure 1), the designated fragment of N gene (180 bp) (Figure 2), and the entire N gene (1260 bp, Supplementary 1) for SARS-CoV-2 isolate Wuhan-Hu-1 (ref. genome), SARS-CoV-2/human/Omicron, SARS-CoV-2/human/B.1.617.2 lineage (Delta variant), SARS-CoV-2/human/P.1 (Gamma variant), SARS-CoV-2/human/Alpha variant, bat SARS-like coronavirus isolate bat-SL-CoVZC45, SARS coronavirus Tor2, and bat coronavirus BM48-31/BGR/2008 to better distinguish the mutations/mismatch along with the PCR products.

MSA through MUSCLE and NCBI Basic Local Alignment Search Tool (BLAST) for nucleotide query (BLASTN; https://blast.ncbi.nlm.nih.gov/Blast.cgi?PROGRAM=blastn&PAGE_TYPE=BlastSearch&LINK_LOC=blasthome) revealed that sequence identities/homologies of E genes in reference to the entire E gene of SARS-CoV-2 Wuhan-Hu-1 (GenBank ID : NC_045512.2, nucleotide seq. position: 26245–26472, 228 bp) were 99.6% (227/228 bp) to SARS-CoV-2/human/Omicron variant (GenBank ID : OM287553.1, nucleotide seq. position: 26170–26397), 100% each to SARS-CoV-2/human/B.1.617.2 lineage (Delta variant; GenBank ID : OK091006.1, nucleotide seq. position: 26218–26445), SARS-CoV-2/human/P.1 (Gamma variant; GenBank ID : MZ427312.1, nucleotide seq. position: 26214–26441), SARS-CoV-2/human/Alpha variant (GenBank ID : MZ888575.1, nucleotide seq. position: 26197–26424), 98.7% (225/228 bp) to bat SARS-like coronavirus isolate bat-SL-CoVZC45 (GenBank ID : MG772933.1, nucleotide seq. position: 26150–26377), 94% (217/231 bp, Gaps: 3/231 (1%)) to SARS coronavirus Tor2 (GenBank ID : NC_004718.3, nucleotide seq. position: 26117–26347), and 91.2% (210/232 bp, Gaps: 5/232 (2%)) to bat coronavirus BM48-31/BGR/2008 (GenBank ID : NC_014470.1, nucleotide seq. position: 26018–26248), respectively.

MSA revealed that 32 nucleotide position mismatches were distinguished in E gene among the 8 GenBank sequences analyzed. We found that the bat coronavirus BM48-31/BGR/2008 (GenBank ID : NC_014470.1) had the highest divergence from the ref. SARS-CoV-2 sequence in the context of E gene homology as this bat coronavirus contributed 27 of 32 nucleotide position mismatches (84%) in E gene. Among SARS-CoV-2 VOCs analyzed, there was only a single nucleotide polymorphism (SNP), which is also a non-synonymous mutation (amino acid alteration) detected at 26195 nucleotide seq. position in E gene of SARS-CoV-2/human/Omicron variant (OM287553.1) (Figure 1).

BLASTN and MSA for PCR amplicon (113 bp) of the E gene fragment as used by [1], taking reference SARS-CoV-2 Wuhan-Hu-1 (NC_045512.2 nucleotide seq. position: 26269–26381, 113 bp size), identified 100% homology each to SARS-CoV-2/human/B.1.617.2 lineage (Delta variant; OK091006.1 nucleotide seq. position: 26242–26354), SARS-CoV-2/human/P.1 (Gamma variant; MZ427312.1 nucleotide seq. position: 26238–26350), SARS-CoV-2/human/Alpha variant (MZ888575.1 nucleotide seq. position: 26221–26333), 99.8% (112/113 bp) homology to SARS-CoV-2/human/Omicron variant (OM287553.1 nucleotide seq. position: 26194–26306), 99.1% (112/113 bp) homology to SARS coronavirus Tor2 (NC_004718.3 nucleotide seq. position: 26141–26253), 98.2% (111/113 bp) homology to bat SARS-like coronavirus isolate bat-SL-CoVZC45 (MG772933.1 nucleotide seq. position: 26174–26286), and 95% (107/113 bp) homology to bat coronavirus BM48-31/BGR/2008 (NC_014470.1 nucleotide seq. position: 26042–26154), respectively.

We distinguished a total of 7 nucleotide position mismatches within 113 nucleotide-long E gene PCR amplicon fragments (6.2%) among the 8 GenBank sequences analyzed. We also observed that the major mismatch (4 of total 7 mismatches = 57%) was contributed by bat coronavirus BM48-31/BGR/2008 (NC_014470.1) in 113 bp long E gene PCR amplicon fragment among the 8 sequences tested. Of 7 mismatches in E gene PCR amplicon fragment, 4 (57%) mismatches were identified in the nucleotide seq. region of forward primer, probe, and reverse primer position. In forward primer, probe, and reverse primer nucleotide seq. region, 3 (75%) nucleotide position mismatches were attributed to bat coronavirus BM48-31/BGR/2008 (NC_014470.1) and 1 (25%) mismatch was constituted by SARS-CoV-2/human/Omicron variant (OM287553.1). While analyzing E gene PCR amplicon fragment, there was a perfect sequence match observed among SARS-CoV-2 VOCs (Alpha, Gamma, Delta) in comparison with ref. sequence (SARS-CoV-2 Wuhan-Hu-1) (Figure 1). None of the primers and probe sets recommended by other WHO-collaborating laboratories consisted of the E gene and did not differentiate screening and confirmation target genes [19].

In reference to the entire N gene of SARS-CoV-2 Wuhan-Hu-1 (GenBank ID : NC_045512.2, nucleotide seq. position: 26245–26472, 228 bp), the sequence identities of the N genes were 98.9% (1247/1260 bp, Gaps: 9/1260 (0.7%)) to SARS-CoV-2/human/Omicron variant (GenBank ID : OM287553.1), 99.7% (1256/1260 bp) to SARS-CoV-2/human/B.1.617.2 lineage (Delta variant; GenBank ID : OK091006.1), 99.5% (1254/1260 bp) to SARS-CoV-2/human/P.1 (Gamma variant; GenBank ID : MZ427312.1), 97.4% (1226/1259 bp) to SARS-CoV-2/human/Alpha variant (GenBank ID : MZ888575.1), 91.1% (1148/1260 bp) to bat SARS-like coronavirus isolate bat-SL-CoVZC45 (GenBank ID : MG772933.1), 88% (1119/1269 bp, Gaps: 9/1269 (0.7%)) to SARS coronavirus Tor2 (GenBank ID : NC_004718.3), and 78% (986/1266 bp, Gaps: 18/1266 (1%)) to bat coronavirus BM48-31/BGR/2008 (GenBank ID : NC_014470.1), respectively.

BLASTN and MSA for PCR amplicon (128 bp) of the N gene fragment as used by [1], taking reference SARS-CoV-2 Wuhan-Hu-1 (NC_045512.2 nucleotide position: 28706–28833, 128 bp size), identified 100% sequence identities each to SARS-CoV-2/human/Omicron variant (OM287553.1 nucleotide position: 28622–28749), SARS-CoV-2/human/B.1.617.2 lineage (Delta variant; OK091006.1 nucleotide position: 28679–28806), SARS-CoV-2/human/P.1 (Gamma variant; MZ427312.1 nucleotide position: 28679–28806), SARS-CoV-2/human/Alpha variant (MZ888575.1 nucleotide position: 28656–28783), 97.7% (125/128 bp) sequence identity and 3 bp mismatch to bat SARS-like coronavirus isolate bat-SL-CoVZC45 (MG772933.1 nucleotide position: 28611–28738), 94.5% (121/128 bp) sequence identity and 7 bp mismatch to SARS coronavirus Tor2 (NC_004718.3 nucleotide position: 28555–28682), and 80.8% (101/125 bp) sequence identity and 24 bp mismatch to bat coronavirus BM48-31/BGR/2008 (NC_014470.1 nucleotide position: 28094–28218), respectively.

We distinguished a total of 31 nucleotide position mismatches within 128 nucleotide-long N gene PCR amplicon fragments (24.2%) among the 8 GenBank sequences analyzed. We also observed that the major mismatches (27 of total 31 mismatches = 87%) were attributed to bat coronavirus BM48-31/BGR/2008 (NC_014470.1), followed (10 of 31 mismatches) by SARS coronavirus Tor2 (NC_004718.3) and (7 of 31 mismatches) by bat SARS-like coronavirus bat-SL-CoVZC45 (MG772933.1). Of 31 mismatches of 128 bp long N gene PCR amplicon fragment, 15 (48%) mismatches were identified in the nucleotide region of forward primer, probe, and reverse primer position. While analyzing MSA of 128 bp long N gene PCR amplicon fragment, there was a perfect sequence match observed among SARS-CoV-2 VOCs (Alpha, Gamma, Delta, Omicron) in comparison with ref. sequence (SARS-CoV-2 Wuhan-Hu-1) (Figure 2).

These analyses signify that both E and N gene primers and probes designed by the Corman group could reliably identify the SARS-CoV-2 strain at that time and the dominant variants that would come at least until early 2022. Most of the heterogeneities in MSA were attributed to bat SARS-like coronavirus, SARS-CoV, and bat coronaviruses. Mutations in the 5' ends of primers and 3′ ends of probes may not significantly hamper PCR efficiency. As E gene was the most conserved among the coronaviruses, the selection of this gene as screening gene was obvious. The use of degenerate nucleotides in the primers and probes or selection and optimization of primer and probe binding sites from more conserved areas could have more correctly screened the novel SARS virus at that time.

China-CDC targeted ORF1ab and N genes and Institut Pasteur, Paris, France, targeted two regions of RdRp gene. Genes targeted by US CDC were two regions of N gene and by the National Institute of Infectious Diseases, Japan, were ORF1ab and S genes for conventional PCR and N gene for RT-PCR. Hong Kong University, China, targeted NSP14 in ORF1b and N genes, and the National Institute of Health, Thailand, used N gene [19].

As of now, no significant problems in the PCR-based diagnosis of COVID-19 have occurred. Concerns and issues related to false-positive tests due to late-Ct value produced by the Corman primers (Eurosurveillance Editorial Team 20210) [20, 21] have been reported. The selection of multiple gene targets can solve this problem and efficiently and precisely detect SARS-CoV-2.

### 3.2. Drifting Variants/VOCs of SARS-CoV-2

Various SARS-CoV-2 variants have been evolving and circulating worldwide since their emergence. Different VOCs have been reported till date. WHO has defined VOCs as “having either increased transmissibility or detrimental epidemiology, or increased virulence or clinical presentation, or decreased effectiveness of public health and social measures or available diagnostics, vaccines and therapeutics” [6]. One of the first variants (not recorded as “variant of concern”) was a D614G mutation, which was first reported in March 2020. The variant was called the “G clade” and was found to spread faster [22]. It represented 10% of global sequenced infection before March 2020 to around 80% by mid-May 2020. Regarding G clade, in vitro study found increased infectivity [22]. In fact, all of the VOCs today are subtypes of the G clade. Since then, the pandemic has been dominated by the Delta variant and recently by the Omicron variant.

Figure 2 shows the increasing prevalence of G clade in 2020 and the domination of the G clade VOCs in 2021. The defining mutations of all of the VOCs are present in spike protein, while mutations in other genes may also affect the biological characteristics of the variants.

**Figure 3:**
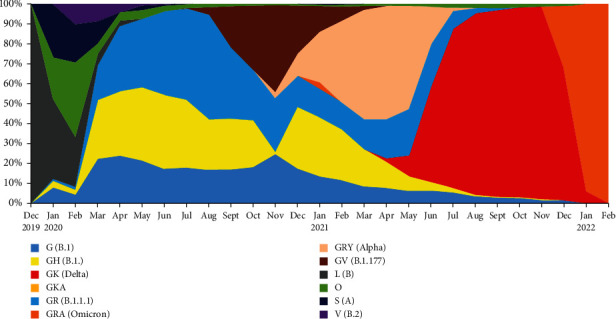
shows the increasing prevalence/distribution of SARS-CoV-2 G clade in 2020 and the domination of the G clade VOCs in 2021.

The major prevalence/distribution of SARS-CoV-2 G clades was observed in 2020, while the domination of the G clade VOC distribution was seen in 2021 (Figure 3). Three VOCs, Alpha, Delta, and Omicron variants, have dominated the SARS-CoV-2 pandemic. The Alpha variant (B.1.1.7) was first detected in the United Kingdom in November 2020 and remained a dominant variant till the emergence of Delta variant. It has 17 mutations, 8 in spike protein among which mutation N501Y enhanced its binding affinity and increased this variant transmissibility by more than 40% of the original strain [25]. The Delta variant (B.1.617.2 lineage), which was first reported from India in December 2021, remained the most prevalent variant till the emergence of the Omicron variant. Delta variant is considered to be 40–60% more transmissible than the Alpha variant [24]. According to the US FDA, N gene and S gene dropouts are not typically seen in Delta variant [10]. As of March 2022, the Omicron variant (BA.1), which was first reported from Botswana and South Africa in November 2021, has become the most dominant variant. The Omicron variant has an N gene with nine-nucleotide deletion from 28370 till 28362 bp, and an S gene with a specific deletion (delE31/R32/S33) can cause gene-targeted failure for N and S gene and reduced test sensitivity [10]. Although the PCR assay/test that has multiple targets may not have a reduction in sensitivity, the defining mutations of all of the VOCs are present in spike protein while mutations in other genes may also affect the biological characteristics of the variants. The loss in sensitivity in PCR diagnosis has occurred for commercial kits targeting the genomic sites where mutations have been emerging. Commercial kits will not have decrease in PCR sensitivity if they are specifically targeting genomic regions where mutations are not detected.

Table 2 demonstrates mutations present in spike proteins of the VOCs. The Sanger sequencing of the given nucleotide regions of the spike proteins may help to identify the strains/variants at a local level. To identify SNPs or mutations in VOCs, cutting-edge molecular PCR assays can be utilized. Optimization to differentiate these variants can prevent the cost of sequencing, but SNP-specific PCR may be cumbersome if there are many variants. Also, this SNP-specific PCR may be easily hampered by any new insignificant SNP near the target SNPs.

The molecular construction of SNP-specific primers and probe targeting genes of interest is essential to efficiently differentiate the drifting variants of SARS-CoV-2, which can identify VOCs.

### 3.3. Rational Selection of PCR Primer and Probe Binding Sites

**Figure 4:**
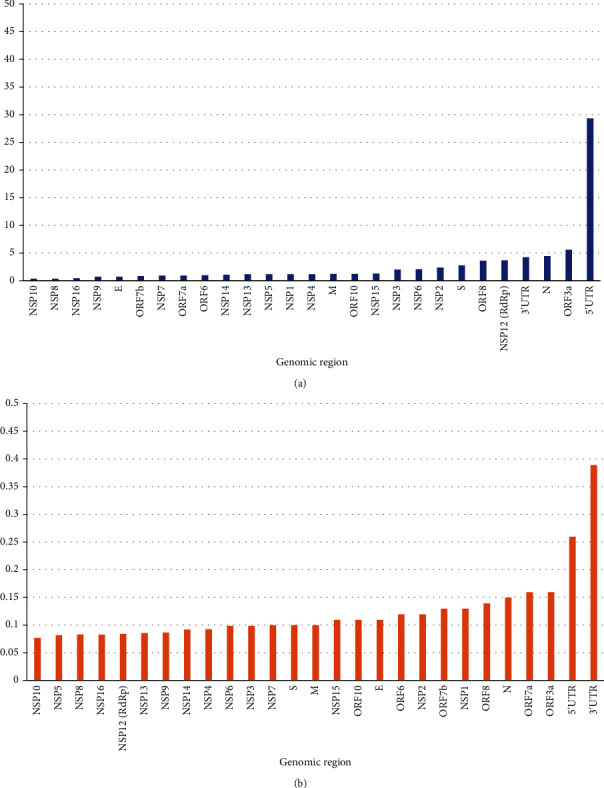
(a). Total SNP counts per nucleotide per 10000 genomes. (b). Unique SNP counts per nucleotide per 10000 genomes.

Reference [15] has analyzed over 48 thousand SARS-CoV-2 genomes till June 26, 2020, deposited them in the GISAID database till June 26, 2020, and found over 350 thousand mutations in the viral genomes [15]. The effect of mutations on qPCR sensitivities has been exemplified in a previous influenza pandemic [25]. The impact of mutations on PCR sensitivity carried at the community- or country-level depends upon two factors: first, the relative propensities of the target gene areas to undergo mutation and, second, the prevalence of such mutated clades/strains in the population [26]. The first scenario is described as prevalent mutations (Table 3A, Figure 4(a)) and the second by unique mutations (Table 3B, Figure 4(b)) in this article. We attempted to identify which genes had lesser mutability and whether it could be used as reliable target sites for PCR.

Based on the number of unique SNPs per nucleotide, we found that NSP10 was the most conserved region (Figure 4), and 3' UTR and 5′ UTR were the least conserved regions with a high tendency to undergo mutation compared with other regions. Similar results were seen when the prevalence of total/prevalent SNPs was compared among different genomic regions with NSP10 being the most conserved and 5′ UTR being the least (Figures 4(a) and 4(b)). In general, nonstructural proteins were more conserved compared with structural ones (Table 3). During the multiplication process of the virus, the whole plus-stranded genomic RNAs are synthesized from minus-strand templates by a single type of replication machinery [9], and thus, the nonstructural genes in open reading frame 1ab and the nonstructural genes in the 3' end of the coronavirus genome should have similar mutation rates. While there could be different factors involved in this phenomenon, one of them could be evolutionary pressure for structural proteins to evolve [27]. Structural proteins are exposed to antibodies in the respiratory mucosa or blood during infection and transmission from one cell to another. The nonstructural proteins help in the intracellular physiology, particularly related to replication and transcription, and thus are unexposed to antibody. Thus, the structural proteins need to evolve to evade antibody-based suppression [27] of infection to new cells. Similarly, based on the results (prevalent and unique mutations) obtained, E protein was the most stable followed by M, N, NSP12ab/RdRp, and S. A similar study carried out in the United States found leader sequence, NSP2, NSP3, RdRp, helicase (NSP13), spike, ORF3a, ORF8, and nucleocapsid proteins to have accumulated mutations during a 4-month period (January to April) in 2020 and few other (NSP7, NSP9, NSP10, NSP11, Envelope, ORF6, and ORF7b proteins) did not accumulate mutation [28]. The study looked into non-synonymous mutations, whereas our study included both synonymous and non-synonymous mutations as both kinds of mutations impact PCR. As found in their study, NSP9 and NSP10 are also among the least mutating genes in our study. Similarly, 5′ UTR leader, ORF3, N, and ORF8 are highly mutating in both of these studies.

Usually, synonymous (or silent) mutations are not responsible for changes in amino acid sequences. Only non-synonymous changes alter the amino acid sequence in proteins, but PCR deals with nucleotide sequences in DNA or cDNA. Thus, both synonymous and non-synonymous changes impact the binding of primers and/or probes. Hence, we used both synonymous and non-synonymous sequences for our analyses.

### 3.4. Rational Primer and Probe Concentration Targeting Genes of Interest in Host Cells

**Figure 5:**
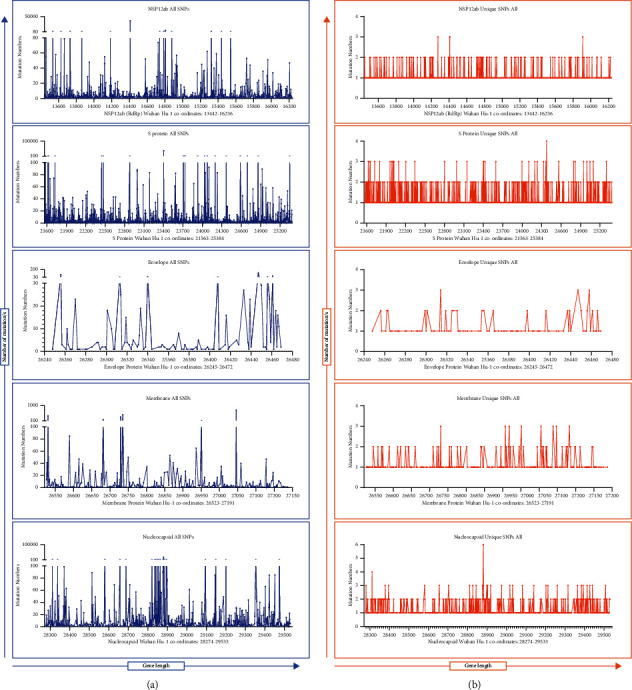
Visual representation of prevalent (a) and unique SNPs (b) in NSP12 (RdRp), S E, M, and N genes.

Transcriptomic analysis of SARS-CoV-2 has repeatedly shown a higher prevalence of reads from the 3' sub-genomic RNAs in the infected host cells [29]. While this could be due to a higher concentration of the sub-genomic RNAs located in the 3′ end, the methodological bias due to sequencing from the 3' end of the genome might have impacted the viral sequence reads [29]. The higher reads in the 3′ region have been supported by translational studies [30]. Reference [31] has iterated that the cellular concentration of plus-stranded RNAs, which are synthesized using minus-strand as a template, is 50- to 100-fold higher than the minus-stranded RNA for coronaviruses (Stanley G) [31]. The transcription mechanism of the coronavirus causes the 3′ end of its genome to have higher reads. The so-called nested sub-genomic structures are formed during the coronavirus transcription process where the genes in the one-third 3' end of the viral genome are translated alone but not transcribed alone. This means, whenever a gene, 3′ to the ORF genes, is transcribed for its translation, all other genes in 3' direction to that particular gene are redundantly transcribed too. Considering that the concentration of each of the plus-stranded translatable sub-genomic RNA units is 50–100 times more than their corresponding minus-strands, this results in a higher number of sequence reads for genes as we go towards the 3′ end of the genome causing the highest number of reads for N gene RNA followed by M, E, and S [29]. This could be the reason why the Ct values for structural genes N and E have better readings (lower Ct values) than that of the RdRp genes [32,33]. Thus, the 3' end sub-genomic RNAs for the structural proteins, which are present at higher concentration in the clinical samples, can be better regions for primer and probe design in terms of better analytical sensitivity. Genetic positions within specific genes may have variable propensities to mutate depending on the exposure of different motifs to the antibody environment or abilities to cope with changes in amino acid combinations. The mutation patterns, prevalent (Figure 5(a)) and unique (Figure 5(b)) along 5′ to 3′ direction for target genes used in qPCR diagnosis (NSP12ab/RdRp, S, E, M, and N), are shown in Figure 5. Visual screening of the mutation status along the nucleotide length of the genes can aid the selection of precise regions of each gene for primer and probe design. One should be careful to interpret that the shown mutation pattern in Figure 5 represents more than 45000 viral genomes, and thus, any one clinical sample is highly unlikely to contain all the mutations.

## 4. Conclusion

For routine point-of-care SARS-CoV-2 confirmatory laboratory diagnosis, rRT-PCR/qPCR is considered as a gold standard technique and is still widely used in the battle against the ongoing pandemic threat posed by the emergence of COVID-19 drifting variants/VOCs. The selection of better PCR target amplicon regions depends upon various factors. It may depend upon whether the investigators want to detect SARS-CoV-2 along with other related viruses. Investigators may also wish to determine and/or discriminate exact variants of interest present in the communities. Evolving new mutation or drifting variants in SARS-CoV-2 genomes may render PCR assay to have variable sensitivities and specificities.

We recommend low mutating structural genes to be used to better discriminate drifting variants of SARS-CoV-2 diagnosis. Recently, the S gene target failure in the UK variant of concern (Alpha strain) strains tested by Applied Biosystems TaqPath RT-PCR COVID-19 Kit was found to be due to 6-nucleotide deletion mutation in the spike gene region targeted by the kit [34]. If a diagnostic kit targets only S gene at the 6-nucleotide deletion region, the diagnostic result could be interpreted as negative for the clinical samples tested. Thus, commercial kits with multiple genetic targets are advisable for the precise diagnosis of COVID-19. While the construction of primers and probes and the product sizes of individual genes in multiplex real-time PCR, in general, are maintained to be of equal or near-equal and shorter lengths (100–150 bp) and of equivalent GC contents among the target amplicons in addition to other factors [35], primers with higher GC contents (40 to 60%) help to prevent mismatch stabilization and ensure stable binding of primers and template. Designing primers to amplify a segment ranging from 60 to 150 bp enhances PCR efficiency. This allows the selection of regions with lower SNP loads within target genes, to design reliable primers and probes. The structural proteins 3′ end sub-genomic RNAs exhibiting relatively higher concentration in clinical samples could be the suitable region for designing primers and probe set for better analytical sensitivity.

The findings of this study revealed that the gene of interest “E gene,” which is the most conserved sequence and highly expressible structural gene of SARS-CoV-2 genomes, needs to be prioritized for the design of primers and probes for PCR-based assays for efficient diagnosis. In addition, SNP-specific binding regions of spike (S gene) protein should be considered for the construction of primers and probes with shorter PCR amplicon size, which enhances the efficiency and precision for VOC differentiation in SARS-CoV-2 diagnosis.

This study recommends the rational primer and probe design targeting the conserved sequence region of E gene, SNP-specific binding regions of spike (S gene) protein, multiple genetic targets with relatively lower mutability and detectable concentration level (ORF7a, ORF7b, etc.), target amplicons with equivalent GC contents and lower SNP/mutation loads, and shorter amplicon size (100–150 bp) to be necessary for the PCR assays to achieve optimal efficiency, sensitivity, and reproducibility in the diagnosis of SARS-CoV-2 variants. However, each primer and probe set designed needs to be evaluated, optimized, and validated prior to being used in routine laboratory diagnosis.

## Figures and Tables

**Table 1:** Sample size and distribution of SARS-CoV-2 genomes on the basis of region for this study.

S. no.	Sample distributed by region	Sample size (*n*)	Sample distribution in percentage (%)
**1**	Africa	514	1.05
**2**	Asia	3340	6.80
**3**	Europe	31818	65.40
**4**	North America	10250	21
**5**	Oceania	2127	4.30
**6**	South America	575	1.10
**7**	Not defined^*∗*^	11	0.02
Total sample size (N)		48635	**100**

**Table 2:** SNP profiles in S gene of SARS-CoV-2 VOCs.

Common name	UK variant		South African variant		Brazilian variant		Indian double mutant		Indian double mutant		Omicron	
Country linked to	UK		South Africa		Brazil		India		India		South Africa	
Pango lineage	B.1.1.7		B.1.351		P.1		B.1.617.2		AY.1		BA.1	
**Variant a/c GISAID**	VUI202012/01 GRY (B.1.1.7)		GH/501Y.v2 (B.1.351)		GR/501Y.V3 (P.1)		**G**	**G**	**G**	**G**	GRA	
WHO names	Alpha	Alpha	Beta	Beta	Gamma	Gamma	Delta	Delta	Delta (B.1.617.2-like) +K417N	Delta (B.1.617.2-like) +K417N	Omicron	Omicron
Mutation/GISAID entry example	EPI_ISL_601443	EPI_ISL_581117	EPI_ISL_678595	EPI_ISL_660222	EPI_ISL_792683	EPI_ISL_792680	EPI_ISL_2131567	EPI_ISL_2441609	EPI_ISL_2171265	EPI_ISL_2479976	EPI_ISL_11457659	EPI_ISL_11655397
Spike L18F			L18F		L18F	L18F						
Spike T19R							T19R	T19R	T19R			
Spike T20N					T20N	T20N						
Spike P26S					P26S	P26S						
Spike A67V											A67V	A67V
Spike H69del	H69del	H69del									H69del	H69del
Spike V70del	V70del	V70del									V70del	V70del
Spike K77T							K77T					
Spike D80A			D80A	D80A								
Spike T95I									T95I		T95I	T95I
Spike D138Y					D138Y	D138Y						
Spike G142D							G142D		G142D		G142D	G142D
Spike V143											V143del	V143del
Spike Y144del	Y144del	Y144del									Y144del	Y144del
Spike Y145del											Y145del	Y145del
Spike E156G										E156G		
Spike 157del									F157del			
Spike R158del									R158del			
Spike R190S					R190S	R190S						
Spike N211del											N211del	N211del
Spike L212I											L212I	L212I
Spike ins214EPE											ins214EPE	ins214EPE
Spike D215G			D215G	D215G								
Spike L242del			L242del	L242del								
Spike A243del			A243del	A243del								
Spike L244del			L244del	L244del								
Spike W258L									W258L	W258L		
Spike G 339D											G339D	G339D
Spike R346K											R346K	R346K
Spike S371L											S371L	S371L
Spike S373P											S373P	S373P
Spike S375F											S375F	S375F
Spike K417T			K417N	K417N	K417T	K417T			K417N	K417N	K417N	K417N
Spike N440K											N440K	N440K
Spike G446S											G446S	G446S
Spike L452R							L452R	L452R	L452R	L452R		
Spike S477N											S477N	S477N
Spike T478K							T478K	T478K	T478K	T478K	T478K	T478K
Spike E484Q			E484K	E484K	E484K	E484K						
Spike E484A											E484A	E484A
Spike Q493R											Q493R	Q493R
Spike G496S											G496S	G496S
Spike Q498R											Q498R	Q498R
Spike N501Y	N501Y	N501Y	N501Y^*∗*^	N501Y	N501Y	N501Y					N501Y	N501Y
Spike Y505H											Y505H	Y505H
Spike T547K											T547K	T547K
Spike A570D	A570D	A570D										
Spike D614G	D614G	D614G	D614G	D614G	D614G	D614G	D614G	D614G	D614G	D614G	D614G	D614G
Spike H655Y					H655Y	H655Y					H655Y	H655Y
Spike N679K											N679K	N679K
Spike P681H	P681H	P681H					P681R	P681R	P681R	P681R	P681H	P681H
Spike A701V			A701V	A701V								
Spike T716I	T716I	T716I										
Spike N764K											N764K	N764K
Spike D796Y											D796Y	D796Y
Spike N856K											N856K	N856K
Spike D950N							D950N	D950N	D950N	D950B		
Spike Q954H											Q954H	Q954H
Spike N969K											N969K	N969K
Spike L981F											L981F	L981F
Spike S982A	S982A	S982A										
Spike T1027I					T1027I	T1027I						
Spike D1118H	D1118H	D1118H										
Spike V1176F					V1176F	V1176F						
^*∗*^Not in the individual database but remarked as defining.												

**Table 3:** Number of prevalent (3A) and unique (3B) SNPs in various target genes of SARS-CoV-2 genomes.

3A				3B			
Genomic regions	Total SNP counts	Region size	Total SNP counts per Nt per 10000 genomes	Genomic region	Unique SNP counts	Region size	Unique SNP counts per Nt per 10000 genomes
NSP10	718	417	0.35	NSP10	158	417	0.078
NSP8	1238	594	0.43	NSP5	365	918	0.082
NSP16	2050	894	0.47	NSP8	239	594	0.083
NSP9	1179	339	0.72	NSP16	361	894	0.083
**E**	837	228	0.75	NSP12 (RdRp)	1140	2795	0.084
ORF7b	574	132	0.89	NSP13	756	1803	0.086
NSP7	1112	249	0.92	NSP9	143	339	0.087
ORF7a	1664	366	0.93	NSP14	706	1581	0.092
ORF6	904	186	0.99	NSP4	679	1500	0.093
NSP14	8351	1581	1.09	NSP6	418	870	0.099
NSP13	10084	1803	1.15	NSP3	2818	5835	0.099
NSP5	5176	918	1.16	NSP7	122	249	0.1
NSP1	3056	540	1.16	S	1897	3811	0.1
NSP4	8696	1500	1.19	M	340	669	0.1
M	4059	669	1.25	NSP15	543	1038	0.11
ORF10	710	117	1.25	ORF10	64	117	0.11
NSP15	6470	1038	1.28	E	125	228	0.11
NSP3	56466	5835	1.99	ORF6	111	186	0.12
NSP6	8697	870	2.06	NSP2	1157	1914	0.12
NSP2	22135	1914	2.38	ORF7b	85	132	0.13
S	51998	3811	2.81	NSP1	351	540	0.13
ORF8	6514	366	3.66	ORF8	256	366	0.14
NSP12 (RdRp)	50441	2795	3.71	N	930	1260	0.15
3′ UTR	4738	229	4.25	ORF7a	277	366	0.16
N	27209	1260	4.44	ORF3a	631	828	0.16
ORF3a	22717	828	5.64	5′ UTR	334	265	0.26
5′ UTR	37872	265	29.38	3′ UTR	433	229	0.39

## Data Availability

Raw data available in the supplementary files in this study were taken from [15] (permission for using data has been sought and granted from the corresponding author), which were originally extracted/downloaded from http://www.GISAID.org. Excel files are available in the supplementary section. GraphPad files used during the analysis in this study are available on request.

## Supplementary Materials

Supplementary Materials Supplementary 1: Multiple sequence alignment (MUSCLE) data/diagram of the entire N gene among analyzed 8 coronavirus sequences (NCBI GenBank IDs) by MEGA11 version 0.1 and is presented. Supplementary 2:: Original raw file downloaded from [15]. It contains all the records of mutations in SARS-CoV-2 genomes studied. Supplementary 3, 4, 5, 6, and 7: spreadsheet files for mutation analyses for NSP12ab, S, E, M, and N genes of SARS-CoV-2 genome, respectively, and are available on request.
