# Supplementary material for: Rational Primer and Probe Construction in PCR-Based Assays for the Efficient Diagnosis of Drifting Variants of SARS-CoV-2
Source: Adv Virol. 2022 May 13;2022:2965666. doi: 10.1155/2022/2965666 (PMC9122727; doi:10.1155/2022/2965666)
Supplement: Supplementary Materials — Supplementary 1: Multiple sequence alignment (MUSCLE) data/diagram of the entire N gene among analyzed 8 coronavirus sequences (NCBI GenBank IDs) by MEGA11 version 0.1 and is presented. Supplementary 2:: Original raw file downloaded from [15]. It contains all the records of mutations in SARS-CoV-2 genomes studied. Supplementary 3, 4, 5, 6, and 7: spreadsheet files for mutation analyses for NSP12ab, S, E, M, and N genes of SARS-CoV-2 genome, respectively, and are available on request. [file 2965666.f1.zip › 2965666.f1/Supplementary 1 (1).doc]

Supplementary 1:

Entire N gene multiple sequence alignment (MUSCLE) by MEGA11 version 0.1

N gene MSA (MUSCLE) by MEGA11 version 0.1. Star (*) signs denote perfect nucleotide position match and empty boxs denote mismatch. Analyzed NCBI GenBank accession IDs for MSA for the entire N gene represent as follows:

1. NC_045512.2: N gene (nucleotide sequence: 28274 – 29533, 1260 bp), SARS-CoV-2 isolate Wuhan-Hu-1,

SARS-CoV-2 ref. genome

2. OM287553.1: N gene (nucleotide sequence: 28199 – 29449, 1251), SARS-CoV-2/human/NLD/EMC-Omicron-1/2021

3. OK091006.1: N gene (nucleotide sequence: 28247 – 29506, 1260 bp), SARS-CoV-2/human/JPN/SARS-CoV-2,

B.1.617.2 lineage, Delta variant/2021

4. MZ427312.1: N gene (nucleotide sequence: 28247 – 29506, 1260 bp), SARS-CoV-2/human/DEU/

SARS-CoV-2_P.1(Gamma)_VeroE6_210419_P3/2021

5. MZ888515.1: N gene (nucleotide sequence: 28225 – 29484, 1259 bp), SARS-CoV-2/human/THA/AFRIMS-COV0087/

Alpha variant/2021

6. MG772933.1: N gene (nucleotide sequence: 28179 – 29438, 1259 bp), Bat SARS-like coronavirus isolate

bat-SL-CoVZC45

7. NC_004718.3: N gene (nucleotide sequence: 28120 – 29388, 1269 bp), SARS coronavirus Tor2,

8. NC_014470.1: N gene (nucleotide sequence: 27665 – 28918, 1254 bp), Bat coronavirus BM48-31/BGR/2008
